# Characterizing and Addressing the Financial, Time, and Administrative Burdens of Cancer and its Care

**DOI:** 10.1007/s11864-025-01358-w

**Published:** 2025-10-17

**Authors:** Kendall Lin, Sana Kagalwalla, Arjun Gupta

**Affiliations:** 1 University of Minnesota Medical School, Minneapolis, MN USA; 2 Division of Hematology, Oncology & Transplantation, University of Minnesota, Minneapolis, MN USA; 3 Division of Hematology, Oncology & Transplantation, University of Minnesota, 420 Delaware Street SE, MMC 480, Minneapolis, MN 55455 USA

**Keywords:** Time toxicity, Cancer, Financial toxicity, Time burdens, Administrative burdens, Logistic burdens

## Abstract

The success of cancer care delivery must be evaluated not only by efficacy outcomes such as overall survival, but also by the experiences of patients and their informal caregivers throughout the course of care. Financial, time, and administrative burdens constitute a triad of non-physical “toxicities’’ that greatly affect patient and caregiver experiences—and outcomes— and should be routinely considered and addressed in oncology practice. While all these burdens have been ever-prevalent, the increasing complexity of cancer care has made these issues more relevant over time. Financial and time burdens started receiving mainstream attention in the oncology community circa 2010 and 2020 respectively. Administrative burdens are currently less well defined but are increasingly recognized and consequential. Each component of the triad does not exist in isolation—financial, time, and administrative burdens can overlap with, interact with, and compound each other. Attempting to address one may worsen another—for example, transferring a medicine prescription and driving to another pharmacy 20 miles away where the co-pay is $50 lower may reduce direct medication out-of-pocket costs, but increase both time and administrative burden. The current piece summarizes the current status of the field with the hope it galvanizes the oncology community to understand and manage these burdens, so patients and caregivers can live their fullest lives.

## Introduction

Advances in oncology have led to significant improvement in overall survival and quality of life, yet the benefits of treatment are often offset by the burdens imposed on patients and caregivers beyond disease itself. Financial toxicity, first conceptualized over a decade ago, reframes the economic consequences of cancer care as a treatment-related adverse effect with measurable impacts on adherence, distress, and survival [1]. More recently, the concept of time toxicity has drawn attention to the lived days consumed by cumulative treatment demands (e.g., travel, waiting, and clinic visits) that may rival or even exceed the months of life gained [2]. Administrative and logistic burdens, encompassing the “hidden work” of navigating insurance, scheduling, billing disputes, and prior authorization, have just recently been recognized as distinct drivers of delay, inequity, and demoralization [3]. Together, these burdens represent non-physical treatment-related toxicities that often overlap and reinforce one another, shaping the cancer experience at the household and system levels. This review synthesizes current knowledge across financial, time, and administrative domains, highlighting commonalities, gaps, and opportunities for intervention to ensure that gains in survival are not undermined by avoidable burdens of care.

## Financial Toxicity

The term *financial toxicity*, coined in 2013, frames the economic burden of cancer care as a treatment-related adverse effect analogous to physical toxicities. It encompasses both objective costs (e.g., out-of-pocket spending, income loss) and the subjective distress these costs impose [1]. Far from a collateral consequence, financial hardship constitutes a true treatment-related toxicity—prognostic for adherence, quality of life, and survival—and often experienced more acutely than objective spending measures suggest [1, 4].

Financial hardship is widespread in cancer care despite insurance coverage, with nearly 40% of patients experiencing financial toxicity [1, 5]. More than half of patients on active treatment are underinsured, often spending ≥ 10% of household income out of pocket, with 42% reporting catastrophic burden [1]. Among survivors, 30–70% continue to experience financial toxicity [6]. This widespread burden has prompted efforts to measure and monitor financial toxicity in cancer care. The Comprehensive Score for Financial Toxicity (COST) is the first validated patient-reported outcome for financial hardship in oncology, demonstrating strong reliability and correlation with quality of life and psychological distress [7]. However, the 12-item measure can be burdensome in practice; therefore, a two-item short form was subsequently developed with preserved concordance to the full scale, providing a practical, low-burden tool for clinical screening [8].

Identifying patients at risk is equally important. Financial toxicity arises from the interplay of patient-, system-, and treatment-level factors. At the patient level, low income, unemployment, minority race or ethnicity, younger age, and female sex consistently predict higher financial burden [5, 7]. Clinical factors such as late-stage diagnosis and frequent hospitalizations also increase costs, while rural residence and inadequate insurance further amplify risk [4, 7, 9]. Health system structures also play a central role. Although the Affordable Care Act reduced medical debt by 34% in expansion states, the growth of high-deductible health plans has simultaneously delayed care and added thousands of dollars in out-of-pocket costs [10]. Even insured patients remain vulnerable as cost-sharing policies (e.g., copayments, deductibles, and coinsurance) shift a greater proportion of treatment expenses to households [11, 12]. At the treatment-specific level, systemic therapies are among the strongest drivers of financial toxicity. Conventional chemotherapy and radiation remain costly, but the advent of targeted therapies, checkpoint inhibitors, and CAR-T has escalated expenditures, with many agents launched at annual prices exceeding $100,000 [4, 11]. Pharmaceutical “spiral pricing,” where each new drug enters at a premium above its predecessor, further amplifies these costs [11]. These dynamics collectively situate financial toxicity not only as an individual vulnerability but as a foreseeable consequence of structural and market forces in modern oncology.

The downstream consequences of financial toxicity are far-reaching. Left unaddressed, economic strain undermines nearly every step of the cancer care continuum. Cost-related nonadherence is common: in a pilot study, nearly one in four patients reported skipping prescriptions, one in five under-dosing medications, and many reduced basic necessities—over 40% cut spending on food or clothing and nearly 70% on leisure, often depleting savings or selling possessions [1]. Financial toxicity independently predicts worse quality of life, with significant correlations observed across validated patient-reported outcome measures [7]. Within two years of diagnosis, 40% of patients lose all assets [10]. Even among survivors, nearly 60% report ongoing indebtedness, and those at the end of life often remain financially devastated despite policy reforms [10]. In a landmark study of more than 230,000 patients with cancer, bankruptcy was 2.6 times more common than in the general population and was associated with a nearly 80% increased risk of mortality (HR 1.79, 95% CI, 1.64–1.96) [13]. Beyond material hardship, financial toxicity manifests as psychological distress and maladaptive coping associated with excess mortality. These effects are most pronounced at diagnosis and at the end of life [4, 6, 14]. Importantly, caregivers also bear this burden, often through absorbing direct expenses and facing employment disruptions [12].

## Time Toxicity

Cancer care consumes not only financial resources but also patients’ and caregivers’ most limited resource—time. *Time toxicity* refers to the cumulative burden of cancer care, encompassing all healthcare-related interactions and ancillary demands (e.g., travel, waiting, clinic visits, acute care, and recovery) that displace time at home and in valued activities [2]. Conceptualized within the last 5 years, time toxicity was framed as an adverse effect analogous to physical and financial toxicities [2]. Where physical and financial toxicities capture treatment-related harms to biological and economic well-being, time toxicity formalizes the treatment-related harm to patients’ and caregivers’ lived time [2, 15]. Many systemic therapies offer only two to three months of survival benefit, yet require a comparable amount of time in treatment, travel, or hospitalization, effectively offsetting the gain and highlighting the need to systematically measure time as a core outcome in cancer care [2, 16].

Contact days, defined as the number of days patients spend receiving healthcare outside the home, serve as a metric for quantifying the time burden imposed by cancer care. They may be contrasted with home days, where no medical encounters occur, to capture how treatment displaces time patients might otherwise spend on personal activities [2]. While not all time in care is inherently negative, measuring contact days provides a standardized proxy for opportunity costs. Across cancer populations, contact days account for a striking proportion of patients’ remaining life. Older adults with cancer spend nearly one in five days in care during the first 6 months after diagnosis [17], and similar proportions are observed among patients with metastatic lung or advanced gastrointestinal cancers, where 20–25% of days alive involve healthcare contact [18–20]. Among those with stage IV non-small cell lung cancer, median survival of 3.5 months translated to nearly one in three days spent in care [21]. The burden of contact days is not constant but follows a U-shaped trajectory, peaking shortly after diagnosis and again near the end of life [19, 21]. Even seemingly brief encounters consume substantial time. Routine laboratory visits average 2 h, while multi-service days frequently exceed 4–6 h [22]. When accounting for travel and caregiver accompaniment, routine visits require a median of 3 or more hours home-to-home, meaning that virtually any ambulatory healthcare encounter can translate into a “lost day” [22]. The impact of time toxicity extends beyond patients, with time loss representing “collateral damage” that reaches caregivers, families, and friends [23].

Metrics that focus only on clinic time underestimate time burden, as waiting, commuting, and recovery may stretch a six-hour infusion into a fully lost day for both patients and caregivers [15]. In a qualitative report, patients and caregivers described cancer care as a full-time job, where even routine appointments become all-day affairs that erase control over daily life [23]. Beyond direct encounters, invisible burdens such as insurance disputes, scheduling, and billing represented a hidden “iceberg” of time loss, often more exhausting than treatment itself [23]. These burdens shape decision-making with patients organically recognizing treatment time as life-consuming. In one study, nearly half of patients with advanced cancer spontaneously raised time costs when weighing therapies, and those who did were twice as likely to decline treatment without survival benefit [24].

The burden of time toxicity is heterogeneous, influenced by tumor biology, treatment modality, comorbidities, and broader social context. In colorectal cancer, cetuximab extended survival by only six weeks yet tripled time-toxic days without increasing overall home time, highlighting how marginal survival benefits can come at significant time cost [25]. Patterns also differed by KRAS status: patients with wild-type tumors accrued both more home and time-toxic days, whereas those with KRAS mutations experienced added toxicity without meaningful gains [25]. Treatment choice strongly influences time toxicity. Broadly, concurrent radiotherapy and cytotoxic chemotherapy impose greater burdens than targeted agents, which are associated with fewer contact days and longer survival [17, 20, 21]. Even within the same disease setting, regimens of equivalent survival may differ: in lymphoma, GDP provided more home days and far fewer hospital days compared with DHAP [26]. Beyond biology and treatment, patient factors also matter. Among older patients with metastatic breast cancer, time toxicity was driven more by comorbidities rather than by age alone, with particularly high burdens observed among those with ≥ 3 comorbidities [27]. Structural inequities compound these disparities, with non-White patients disproportionately experiencing even greater time costs [15].

## Administrative and Logistic Burdens

While financial and time toxicities have gained recognition as central challenges in oncology, administrative and logistic burdens represent a more recent domain of exploration. These burdens encompass a wide spectrum of tasks—from scheduling appointments, attending visits, and managing medications to navigating pharmacy runs, insurance paperwork, transportation, and consultations—that cumulatively erode patients’ and caregivers’ time and energy [28]. Many of these responsibilities, particularly home-based administrative tasks, remain largely invisible yet constitute major sources of distress [28]. Herd and Moynihan conceptualize administrative burden as comprising three domains: learning costs (such as deciphering insurance coverage, provider networks, and medical bills), compliance costs (including prior authorizations, billing disputes, and extensive paperwork), and psychological costs (stress, anxiety, and demoralization) [3]. Within cancer care, these burdens often rival financial barriers; indeed, administrative complexity can block access to guideline-concordant treatment at rates comparable to cost-related obstacles [29].

Despite the centrality of administrative challenges in oncology, there is no gold-standard tool for quantifying this burden with existing studies relying on patient-reported surveys or proxy system metrics. Of the various administrative barriers, prior authorization has emerged as perhaps the clearest and most quantifiable driver of burden, with patient-reported surveys consistently documenting its disruptive effects. Although initially intended to limit unnecessary care, it frequently delays or prevents patients from accessing evidence-based treatments [30]. Nearly 70% of patients encounter prior authorization-related delays, with one-third waiting a month or more for cancer care, and 22% ultimately never receiving the therapy their oncologist recommended [30]. Even seemingly modest policy changes have measurable consequences. The introduction of a new PA requirement for an established drug increased discontinuation odds more than sevenfold and extended refill intervals by nearly 10 days [31]. Insurers often rely on denials and PA restrictions to constrain utilization; however, improper denials were found in over half of all Medicare Advantage contracts [3]. Almost two-thirds of patients report having to navigate insurers on their own, with one in five spending more than 11 h on paperwork, appeals, and phone calls [30]. These efforts are not simply inconvenient; they wear away trust and amplify distress. Following prior authorization experiences, 89% of patients reported decreased trust in their insurer and 83% in the broader health system, while anxiety levels nearly doubled compared with baseline [30]. The impact reverberates through the workforce. Each PA submission costs insurers an estimated $40–50 and providers $20–30 in processing, without direct benefit to patients and ultimately driving higher patient costs [32]. Physicians lose roughly three hours weekly to insurance paperwork, equivalent to three full workweeks annually, while nurses and clerical staff spend 19 and 36 h per week respectively—time diverted from patient support and coordination [33]. Nurses frequently characterize these tasks as pointless and repetitive, with implications for burnout and compromised care [34]. Cumulatively, these inefficiencies translate to an estimated $21.6 billion in lost worker time annually [3].

Administrative complexity encompasses a wide range of tasks that extend beyond insurer interactions. Nearly three-quarters of insured adults report engaging in other healthcare-related administrative tasks, with one in four delaying or forgoing care as a result [29]. Scheduling and information-seeking are also widespread burdens; however, complex administrative tasks such as billing disputes, PA requests, and premium management are disproportionately harmful, with up to 45% of patients forgoing care as a result [29]. Vulnerable groups, such as those with rural residence, low income, limited education, disability, immigrant status, working parent responsibilities, and limited social support, experience these burdens most acutely [28]. Caregivers also absorb a parallel share of this burden, often responsible for transportation, appointment coordination, and managing treatment side effects [28]. These invisible responsibilities magnify stress across households, deepening the logistical toll of cancer care and straining the networks patients depend on most.

## Addressing Financial, Time, and Administrative Burdens

Mitigating financial toxicity requires structured, oncology-specific interventions that extend beyond recognition of the problem. Routine screening with validated tools such as the Comprehensive Score for Financial Toxicity (COST) can help identify at-risk individuals early, though uptake remains limited and particularly underdeveloped for caregivers and older adults [4, 14, 35–37]. Embedding financial counseling early in oncology care, through social worker- or nurse navigator-led programs, enables timely referral and direct connection to assistance for prescriptions, insurance navigation, food support, and debt management [14, 35, 37]. Dedicated financial navigators embedded within cancer centers can also provide financial literacy support, facilitate applications for aid, and help patients negotiate payment plans [14, 35, 37]. Oncologists should also take a more active role in cost-conscious care by consolidating visits to reduce travel demands, eliminating low-value encounters without clear benefit, selecting lower-cost but equally effective regimens, and ensuring transparent discussions about the financial implications of clinical trial participation [14, 37]. Emerging delivery innovations in telemedicine and decentralized care, alongside digital tools like mobile applications and integration of patient-reported outcomes, may provide scalable means for ongoing monitoring and communication [36]. At the systems level, durable solutions demand employer, insurer, and policy reforms: cost caps, price transparency, oral chemotherapy parity, coverage for non-medical expenses, job accommodations, and inclusion of financial hardship as a quality metric [6, 35, 37].

Addressing time toxicity requires both systematic measurement and intentional redesign of care delivery. Prospective reporting of contact days should become routine in clinical trials, offering an additional endpoint to guide shared decision-making and serving as a “tie-breaker” when efficacy is comparable [2, 15, 20, 23]. Such reporting provides patients with realistic expectations about treatment-related time demands and allows clinicians to tailor regimens accordingly [17, 23]. Importantly, clinical time is not uniformly burdensome. Some patients derive reassurance from frequent contact, while others find it disruptive, highlighting the value of objective reporting to align treatment with individual priorities [23]. Redesigning care structures offers practical avenues to reduce avoidable time burden. Nearly two-thirds of scans, tests, or treatments occur on separate days, which amplifies logistical strain [38]. Centralized scheduling systems and advanced coordination of labs may help reduce redundant visits and waiting time. Other strategies to meaningfully reduce time-toxic days include consolidating visits, shifting from weekly to biweekly dosing schedules, and expanding use of oral or subcutaneous regimens that reduce infusion chair time [2, 23, 25]. Home-based approaches, such as telemedicine, nurse-administered infusions, or hydration therapy delivered at home, can further minimize travel demands [2, 23]. At a broader level, framing time toxicity as a recognized adverse effect of treatment, analogous to financial or physical toxicities, provides a shared language and actionable levers for policy, research, and clinical reform [23].

Efforts to mitigate administrative and logistic burdens in cancer care span legislative reform, care redesign, and supportive services. At the policy level, bipartisan legislation such as the *Improving Seniors’ Timely Access to Care Act* (2022) and the recently reintroduced *Reducing Medically Unnecessary Delays in Care Act* (2025) reflects growing recognition of prior authorization (PA) as a driver of delay and clinician frustration [39, 40]. Yet, despite broad support, these measures have repeatedly stalled short of meaningful implementation. Within health systems, redesign of PA teams offers more immediate gains: one radiation oncology program that integrated clinical experts (e.g., dosimetrists) reduced appeal times from 30 to 18 days, increased overturned denials by 56%, and enabled 37% more patients to receive timely treatment. Provider burden fell markedly, with physicians handling only 6% of peer-to-peers, while staff efficiency gains allowed a 44% reduction in team size and a 34% cut in personnel costs [41]. Parallel efforts address logistical strain through consolidated scheduling, home- or virtual-based care, and improved coordination [28]. Navigation services, now reimbursable by Medicare and private insurers and covering more than 150 million Americans, have demonstrated a 60% reduction in surgical oncology wait times [42]. Evidence also supports navigation’s role in improving cancer screening uptake, reducing delays from screening to diagnosis and treatment, enhancing quality of life and satisfaction, and, in some contexts, achieving cost-effectiveness [42]. Beyond navigation, medical-legal partnerships represent an emerging intervention to relieve administrative barriers. The *Colorectal Cancer Legal and Administrative Burden Support (COLLABS)* trial tested free legal services for patients with colorectal cancer, targeting insurance disputes, employment protections, and debt relief, and demonstrated the potential to reduce out-of-pocket costs and psychosocial distress [35, 43]. Although efforts are advancing, no standardized tool yet exists to capture the full span of administrative burden, underscoring the need for validated measures and future trials to guide interventions.

These three domains of burden are tightly interwoven, with strain in one area often magnifying pressures in the others. For example, navigating prior authorizations or insurance denials entails significant administrative effort, consumes substantial time through repeated calls and delays, and can impose considerable financial hardship when coverage is denied or deferred [44]. Additionally, efforts to ease one burden may intensify another. In one study, patients who sought to minimize out-of-pocket costs by filling prescriptions at the lowest-cost pharmacies could save only $19 across five supportive care medications yet doing so required over an hour of additional driving—an exchange of financial relief for substantial time and logistic burden [45, 46]. Moreover, pharmacies that were cheapest at one point often became far more expensive within months, forcing patients to re-check prices and transfer prescriptions, compounding administrative work and creating vulnerabilities, as fragmented pharmacy use limits oversight and increases the risk of missed drug-drug interactions [45, 46]. Taken together, these dynamics highlight the need for approaches that address burdens in an integrated rather than siloed manner.

Across financial, time, and administrative domains, several cross-cutting strategies emerge. Routine screening for hardship, whether financial, time, or logistic, remains underutilized but essential for early identification and timely intervention [2, 35, 36]. Navigation services stand out as a unifying solution: financial navigators, care coordinators, and medical-legal partnerships consistently demonstrate improvements in access, timeliness, and patient-centered outcomes while reducing avoidable utilization [35, 41, 42]. Policy reform is similarly indispensable. Legislative efforts to cap costs, enforce parity protections, and streamline prior authorization provide system-level safeguards against financial and administrative strain [35, 37, 39, 40]. Parallel reimbursement reforms and care redesign strategies, such as visit consolidation, telehealth, and home-based care, can further reduce all three burdens [23, 38]. Finally, embedding burden reporting into clinical trials and routine care provides a framework to balance efficacy with lived experience [2, 20, 23]. Together, these strategies provide an opportunity to treat financial, time, and administrative burdens proactively at both the bedside and policy level.

## Conclusion

Financial, time, and administrative burdens have emerged as critical, treatment-related toxicities that shape the lived experience of cancer care. While each domain carries distinct challenges, they often intersect, exacerbating distress, impairing adherence, and undermining the value of therapeutic advances. Evidence now supports routine screening for financial hardship, incorporation of contact days into clinical endpoints, and redesign of administrative processes as actionable steps toward alleviating these burdens. Navigation services, care coordination, and policy reforms offer cross-cutting solutions, but durable progress will require integration across clinical practice, health systems, and legislation. Reducing these avoidable toxicities is not ancillary to oncology but central to its mission: ensuring that patients and caregivers not only survive longer but also retain the resources, time, and dignity to live better.

## Key References

- Zafar SY, Peppercorn JM, Schrag D, et al. The Financial Toxicity of Cancer Treatment: A Pilot Study Assessing Out-of-Pocket Expenses and the Insured Cancer Patient’s Experience. *Oncologist*. 2013;18(4):381–390. doi:10.1634/theoncologist.2012-0279.
  - This reference is of outstanding importance because it coined the term “financial toxicity” and demonstrated that even insured patients on active treatment faced catastrophic costs leading to medication underuse and sacrifices in basic living needs.
- Gupta A, Eisenhauer EA, Booth CM. The Time Toxicity of Cancer Treatment. J Clin Oncol. 2022;40(15):1611–1615. doi:10.1200/JCO.21.02810.
  - This reference is of outstanding importance because it established “time toxicity” as a treatment-related adverse effect in oncology, showing that many therapies consume a comparable amount of time as the survival they confer, thereby reframing patient time as a critical endpoint.
- Herd P, Moynihan D. Health care administrative burdens: Centering patient experiences. Health Serv Res. 2021;56(5):751–754. doi:10.1111/1475-6773.13858.
  - This reference is of outstanding importance because it defined the concept of “administrative burdens” through a patient-centered framework and highlighted their role in delaying or preventing access to care.

## Data Availability

No datasets were generated or analysed during the current study.
